# Supplementary material for: Highly Oxygenated Triterpenoids and Diterpenoids from Fructus Rubi (Rubus chingii Hu) and Their NF-kappa B Inhibitory Effects
Source: Molecules. 2021 Mar 29;26(7):1911. doi: 10.3390/molecules26071911 (PMC8037097; doi:10.3390/molecules26071911)

Supporting Information for

Highly Oxygenated Triterpenoids and Diterpenoids from Fructus Rubi (*Rubus Chingii* Hu) and Their NF-kappa B Inhibitory Effects

Jiang Wan [1,†], Xiao-Juan Wang [1,†], Nan Guo [1], Xi-Ying Wu [1], Juan Xiong [1], Yi Zang [2], Chun-Xiao Jiang, [3] Bing Han [1,*], Jia Li [2,*], and Jin-Feng Hu [1,3*]

[1] Minhang Hospital & School of Pharmacy, Fudan University, Shanghai 201199, PR China; 18111030021@fudan.edu.cn (J.W.); 18221765046@163.com (X.-J.W.); gnguoguo@163.com (N. G.); wxyifd@163.com (X.-Y.W.); jxiong@fudan.edu.cn (J.X.); hbshcn@163.com (B. H.); jfhu@fudan.edu.cn (J.-F.H)

[2] State Key Laboratory of Drug Research, Shanghai Institute of Materia Medica, Chinese Academy of Science, shanghai 201203, PR China;
yzang@simm.ac.cn (Y.Z.); jli@simm.ac.cn (J.L.)

[3] Institute of Natural Medicine and Health Products, School of Advance Study, Zhejiang Provincial Key Laboratory of Plant Ecology and Conservation, Taizhou University, Taizhou 318000, Zhejiang, PR China.
jiangchx15@tzc.edu.cn (C.-X. J)

* Correspondence: jfhu@fudan.edu.cn (J.-F.H); hbshcn@163.com (B. H.); Tel.: +86-21-51980172 (J.-F.H)

† These authors contributed equally to this work.

Contents of supporting information	page
Spectroscopic data for the known compounds **4**–**20** and **22**–**25**	1–4
NMR data and HR-ESIMS for compound **1**	5–7
NMR data and HR-ESIMS for compound **2**	8–10
NMR data and HR-ESIMS for compound **3**	10–13
NMR data and HR-ESIMS for compound **21**	14–16

Spectroscopic data for the known compounds 4–20 and 22–25

Fupenzic acid (**4**): white, amorphous powder; ^{1}H NMR (CD_3OD, 400 MHz): δ 0.88 (3H, s, Me-26), 0.93 (3H, d, J = 6.4 Hz, Me-30), 1.11 (3H, s, Me-24), 1.18 (3H, s, Me-23), 1.20 (3H, s, Me-29), 1.22 (3H, s, Me-25), 1.35 (3H, s, Me-27), 1.74 (1H, br d, J = 11.3 Hz, H-5), 2.00 (1H, dd, J = 10.4, 5.6 Hz, H-9), 2.53 (1H, s, H-18), 5.35 (1H, dd, J = 3.8, 3.1 Hz, H-12), 6.28 (1H, s, H-1); ESI-MS: 483 $[M - H]^-$.

2α,3α,19α-trihydroxy-urs-12-en-28-oic acid (**5**): white, amorphous powder, ^{1}H NMR (C_5D_5N, 400 MHz): δ 0.89 (3H, s, Me-26), 0.97 (3H, s, Me-24), 1.10 (3H, s, Me-25), 1.10 (3H, d, J =5.5 Hz, Me-30), 1.26 (3H, s, Me-23), 1.41 (3H, s, Me-29), 1.64 (3H, s, Me-27), 3.03 (1H, s, H-18), 3.76 (1H, br s, H-3), 4.30 (1H, br d, J = 10.0 Hz, H-2), 5.58 (1H, br s, H-12).

2α,3α,23-trihydroxy-urs-12-en-28-oic acid (**6**): white, amorphous powder, ^{1}H NMR (C_5D_5N, 400 MHz): δ 0.86 (3H, s, Me-24), 0.90 (3H, d, J = 5.7 Hz, Me-30), 0.91 (3H, s, Me-25), 0.95 (3H, d, J = 5.7 z, Me-29), 0.98 (3H, s, Me-26), 1.12 (3H, s, Me-27), 2.62 (1H, d, J = 11.2 Hz, H-18), 3.76 (1H, d, J = 10.4 Hz, H-23), 3.93 (1H, d, J = 10.4 Hz, H-23), 4.16 (1H, br s, H-3), 4.29 (1H, br dd, J = 10.6 Hz, H-2), 5.45 (1H, br s, H-12).

2α,19α-dihydroxy-3-oxo-urs-12-en-28-oic acid (**7**): white, amorphous powder, ^{1}H NMR (C_5D_5N, 400 MHz): δ 0.99 (3H, s, Me-24), 1.10 (3H, d, J = 5.5 Hz, Me-30), 1.12 (6H, s, Me-25 and Me-26), 1.21 (3H, s, Me-23), 1.25 (1H, br d, J = 10.2 Hz, H-5), 1.42 (3H, s, Me-29), 1.65 (3H, s, Me-27), 1.87 (1H, dd, J = 9.5, 8.8 Hz, H-9), 2.48 (1H, dd, J = 12.9, 6.3 Hz, H-1), 3.04 (1H, s, H-18), 4.82 (1H, dd, J = 12.9, 6.3 Hz, H-2), 5.56 (1H, dd, J = 3.9, 3.1 Hz, H-12), 5.18 (1H, br s, OH-2).

2α,3β,19α,24-tetrahydroxyurs-12-en-28-oic acid (**8**): white, amorphous powder, ^{1}H NMR (CD_3OD, 400 MHz): δ 0.77 (3H, s, Me-23), 0.93 (3H, d, J = 6.5 Hz, H-30), 0.99 (3H, s, Me-26), 1.19 (3H, s, Me-29), 1.24 (3H, s, Me-25), 1.34 (3H, s, Me-27), 2.50 (1H, s, H-18), 3.06 (1H, d, J = 9.4 Hz, H-3), 3.40 (1H, d, J = 11.0 Hz, H-24), 3.79 (1H, ddd, J = 10.8, 9.4, 4.5 Hz, H-2), 4.04 (1H, d, J = 11.0 Hz, H-24), 5.29 (1H, br s, H-12).

1β,3β,19α-trihydroxy-2-oxo-urs-12-en-28-oic acid (**9**): white, amorphous powder, ^{1}H NMR (CD_3OD, 400 MHz): δ 0.71 (3H, s, Me-24), 0.82 (3H, s, Me-26), 0.83 (3H, s, Me-25), 0.95 (3H, d, J = 7.0 Hz, H-30), 1.19 (3H, s, Me-29), 1.22 (3H, s, Me-23), 1.42 (3H, s, Me-27), 2.52 (1H, s, H-18), 4.06 (1H, d, J = 1.2 Hz, H-3), 4.12 (1H, d, J = 1.2 Hz, H-1), 5.31 (1H, dd, J = 3.6, 3.2 Hz, H-12); ^{13}C NMR (CD_3OD, 150 MHz): δ 85.6 (C-1), 212.1 (C-2), 82.3 (C-3), 46.4 (C-4), 52.6 (C-5), 19.3 (C-6), 33.9 (C-7), 41.9 (C-8), 49.1 (C-9), 50.1 (C-10), 27.7 (C-11), 130.3 (C-12), 138.9 (C-13), 42.6 (C-14), 29.7 (C-15), 26.6 (C-16), 48.5 (C-17), 55.0 (C-18), 73.6 (C-19), 43.0 (C-20), 27.3 (C-21), 39.0 (C-22), 29.3 (C-23), 17.3 (C-24), 12.4 (C-25), 16.8 (C-26), 24.8 (C-27), 182.2 (C-28), 27.0 (C-29), 16.6 (C-30).

3β,19α-dihydroxy-2-oxo-urs-12-en-28-oic acid (**10**): white, amorphous powder, ^{1}H NMR (CD_3OD, 400 MHz): δ 0.72 (3H, s, Me-24), 0.80 (3H, s, Me-26), 0.88 (3H, s, Me-25), 0.92 (3H, d, J = 6.9 Hz, H-30), 1.18 (3H, s, Me-23), 1.19 (3H, s, Me-29), 1.40 (3H, s, Me-27), 2.24 (1H, d, J = 12.1 Hz, H-1a), 2.33 (1H, d, J = 12.1 Hz, H-1b), 2.51 (1H, s, H-18), 3.99 (1H, s, H-3), 5.29 (1H, dd, J = 3.4, 3.2 Hz, H-12).

2α,3α,19α,23-tetrahydroxy-olean-12-en-28-oic acid (**11**): white, amorphous powder, ^{1}H NMR (CD_3OD, 400 MHz): δ 0.77 (3H, s, Me-23), 0.79 (3H, s, Me-26), 0.94 (3H, s, Me-30), 0.97 (3H, s, Me-29), 1.01 (3H, s, Me-25), 1.32 (3H, s, Me-27), 3.06 (1H, d, J = 4.4 Hz, H-18), 3.26 (1H, d, J = 4.4 Hz, H-19), 3.39 (1H, d, J = 11.1 Hz, H-23), 3.56 (1H, d, J = 11.1 Hz, H-23), 3.63 (1H, br s, H-3), 3.89 (1H, dd, J = 11.5, 4.1 Hz, H-2), 5.33 (1H, br s, H-12); ^{13}C NMR (CD_3OD, 150 MHz): δ 42.7 (C-1), 67.3 (C-2), 78.9 (C-3), 42.0 (C-4), 44.3 (C-5), 19.1 (C-6), 34.0 (C-7), 40.8 (C-8), 48.5 (C-

9), 39.3(C-10), 24.9 (C-11), 124.7 (C-12), 144.7 (C-13), 42.5 (C-14), 29.4 (C-15), 28.6 (C-16), 46.7 (C-17), 45.2 (C-18), 82.5 (C-19), 36.0 (C-20), 29.5 (C-21), 33.5 (C-22), 71.3 (C-23), 17.8 (C-24), 17.1 (C-25), 17.5 (C-26), 25.2 (C-27), 182.3 (C-28), 29.4 (C-29), 25.1 (C-30). ESI-MS: 499 $[M-H]^-$.

2α,3α,23-trihydroxy-urs-12-en-28-oic acid (**12**): white, amorphous powder, ^{1}H NMR (C_5D_5N, 400 MHz): δ 0.86 (3H, s, Me-24), 0.89 (3H, s, Me-25), 0.97 (3H, s, Me-26), 1.00 (3H, s, Me-30), 1.03 (3H, s, Me-30), 1.19 (3H, s, Me-27), 3.28 (1H, dd, J = 13.7, 4.6 Hz, H-18), 3.76 (1H, d, J = 10.4 Hz, H-23), 3.93 (1H, d, J = 10.4 Hz, H-23), 4.16 (1H, br s, H-3), 4.30 (1H, br dd, J = 10.6 Hz, H-2), 5.46 (1H, br s, H-12); ^{13}C NMR (C_5D_5N, 150 MHz): δ 42.2 (C-1), 66.2 (C-2), 78.9 (C-3), 41.8 (C-4), 43.5 (C-5), 18.2 (C-6), 33.1 (C-7), 39.8 (C-8), 48.0 (C-9), 38.4 (C-10), 23.6 (C-11), 122.4 (C-12), 144.8 (C-13), 42.5 (C-14), 28.1 (C-15), 23.7 (C-16), 46.6 (C-17), 41.8 (C-18), 46.3 (C-19), 30.8 (C-20), 34.1 (C-21), 32.8 (C-22), 71.2 (C-23), 17.7 (C-24), 16.9 (C-25), 17.5 (C-26), 26.1 (C-27), 180.2 (C-28), 33.1 (C-29), 23.8 (C-30).

Arjunic acid (**13**): white, amorphous powder, ^{1}H NMR (C_5D_5N, 400 MHz): δ 1.02 (3H, s, Me-26), 1.06 (3H, s, Me-25), 1.08 (3H, s, Me-24), 1.11 (3H, s, Me-30), 1.19 (3H, s, Me-29), 1.27 (3H, s, Me-23), 1.64 (3H, s, Me-27), 3.39 (1H, d, J = 9.6 Hz, H-3), 3.61 (1H, d, J = 5.5 Hz, H-19), 3.63 (1H, d, J = 5.5 Hz, H-18), 4.11 (1H, ddd, J = 10.3, 9.3, 4.6 Hz, H-2), 5.55 (1H, br s, H-12); ^{13}C NMR (C_5D_5N, 150 MHz): δ 47.5 (C-1), 68.5 (C-2), 83.7 (C-3), 39.8 (C-4), 55.9 (C-5), 18.9 (C-6), 33.6 (C-7), 40.0 (C-8), 48.4 (C-9), 38.6 (C-10), 24.2 (C-11), 123.3 (C-12), 144.8 (C-13), 42.1 (C-14), 29.1 (C-15), 24.7 (C-16), 46.0 (C-17), 44.8 (C-18), 81.1 (C-19), 35.7 (C-20), 28.3 (C-21), 29.3 (C-22), 28.8 (C-23), 16.7 (C-24), 17.5 (C-25), 17.5 (C-26), 24.7 (C-27), 180.7 (C-28), 29.3 (C-29), 24.7 (C-30); ESI-MS: 511 $[M+Na]^+$, 487 $[M-H]^-$.

2α,3β,19α,24-tetrahydroxyolean-12-en-28-oic acid (**14**): white, amorphous powder, ^{1}H NMR (CD_3OD, 400 MHz): δ 0.77 (3H, s, Me-23), 0.95 (3H, s, Me-26), 0.98 (3H, s, Me-30), 1.00 (3H, s, Me-25), 1.25 (3H, s, Me-29), 1.32 (3H, s, Me-27), 3.07 (1H, d, J = 3.8 Hz, H-18), 3.27 (1H, d, J = 3.8 Hz, H-19), 3.31 (1H, d, J = 10.5 Hz, H-3), 3.42 (1H, d, J = 11.1 Hz, H-24), 3.80 (1H, ddd, J = 10.5, 10.5, 4.5 Hz, H-2), 4.05 (1H, d, J = 11.1 Hz, H-24), 5.34 (1H, dd, J = 3.5, 3.3 Hz, H-12); ^{13}C NMR (CD_3OD, 150 MHz): δ 47.7 (C-1), 69.9 (C-2), 86.0 (C-3), 44.4 (C-4), 57.3 (C-5), 20.0 (C-6), 34.2 (C-7), 40.7 (C-8), 49.6 (C-9), 39.2 (C-10), 28.7 (C-11), 123.6 (C-12), 144.7 (C-13), 42.6 (C-14), 29.5 (C-15), 25.0 (C-16), 46.7 (C-17), 45.1 (C-18), 82.4 (C-19), 36.0 (C-20), 28.6 (C-21), 34.0 (C-22), 23.7 (C-23), 66.2 (C-24), 17.6 (C-25), 17.4 (C-26), 25.0 (C-27), 182.3 (C-28), 29.4 (C-29), 25.1 (C-30).

Cucurbitacin D (**15**): white, amorphous powder, ^{1}H NMR ($CDCl_3$, 400 MHz): δ 0.99 (3H , s, Me-18), 1.09 (3H , s, Me-19), 1.29 (3H , s, Me-28), 1.35 (3H , s, Me-29), 1.36 (3H , s, Me-30), 1.39 (6H , s, Me-26 and Me-27), 1.43 (3H , s, Me-21), 1.86 (1H, dd, J = 12.9, 7.7 Hz, H-15), 1.96 (1H, m, H-7), 1.98 (1H, br d, J = 8.0 Hz, H-8), 2.33 (1H, m, H-1), 2.42 (1H, br dd, J = 19.0 Hz, H-7), 2.57 (1H, d, J = 7.0 Hz, H-17), 2.71 (1H, d, J = 15.7 Hz, H-12), 2.74 (1H, br d, J = 12.2 Hz, H-10), 3.26 (1H, d, J = 15.7 Hz, H-12), 4.40 (1H, dd, J = 8.5, 7.0 Hz, H-16), 4.44 (1H, d, J = 11.7, 4.7 Hz, H-2), 5.79 (1H, br s, H-6), 6.69 (1H, d, J = 15.3 Hz, H-23), 7.12 (1H, d, J = 15.3 Hz, H-24); ESI-MS: 539 $[M+Na]^+$.

3α,16α,20(R),25-tetrahydroxy-cucurbita-5,23-dien-2,11,22-trione (**16**): white, amorphous powder, ^{1}H NMR ($CDCl_3$, 400 MHz): δ 0.78 (3H, s, Me-28), 0.94 (3H, s, Me-18), 1.15 (3H, s, Me-30), 1.22 (3H, s, Me-19), 1.30 (3H, s, Me-29), 1.32 (3H, s, Me-26), 1.32 (3H, s, Me-27), 1.36 (3H, s, Me-21), 1.85 (1H, dd, J = 13.2, 7.8 Hz, H-15), 1.99 (1H, br dd, J = 15.7 6.0 Hz, H-7β), 2.01 (1H, br d, J = 7.4 Hz, H-8), 2.21 (1H, dd, J = 13.2, 12.9 Hz, H-1a), 2.39 (1H, dd, J = 13.2, 5.0 Hz, H-1b), 2.52 (1H, d, J = 7.5 Hz, H-17), 2.62 (1H, d, J = 15.3 Hz, H-12a), 2.72 (1H, dd, J = 12.9, 5.0 Hz, H-10),

3.11 (1H, d, J = 15.3 Hz, H-12b), 3.88 (1H, s, H-3), 4.30 (1H, br dd, J = 7.8, 7.5 Hz, H-16), 5.91 (1H, br d, J = 6.0 Hz, H-6), 6.61 (1H, d, J = 15.3 Hz, H-23), 7.07 (1H, d, J = 15.3 Hz, H-24); ESI-MS: 539 $[M + Na]^+$.

Cucurbitacin B (**17**): white, amorphous powder, ^{1}H NMR (Acetone-d_6, 400 MHz): δ 0.92 (3H, s, Me-18), 1.02 (3H, s, Me-30), 1.29 (3H, s, Me-28), 1.32 (3H, s, Me-29), 1.40 (3H, s, Me-21), 1.44 (3H, s, Me-19), 1.51 (3H, s, Me-27), 1.56 (3H, s, Me-26), 1.83 (1H, dd, J = 13.1, 8.7 Hz, H-15), 1.97 (1H, m, H-7a), 1.97 (3H, s, H-CO$\underline{CH_3}$), 2.11 (1H, m, H-1), 2.40 (1H, m, H-7b), 2.51 (1H, d, J = 14.5 Hz, H-12a), 2.66 (1H, d, J = 7.2 Hz, H-17), 3.01 (1H, br d, J = 12.9 Hz, H-10), 3.40 (1H, d, J = 14.5 Hz, H-12b), 4.46 (1H, br dd, J = 8.7, 7.2 Hz, H-16), 4.56 (1H, dd, J = 12.9, 5.9 Hz, H-2), 5.82 (1H, br d, J = 5.5 Hz, H-6), 6.79 (1H, d, J = 15.7 Hz, H-23), 6.99 (1H, d, J = 15.7 Hz, H-24); ESI-MS: 581 $[M + Na]^+$, 603 $[M + HCOO^-]^-$.

2,16α,20(R),25-tetrahydroxy-cucurbita-1,5,23-trien-3,11,22-trione (**18**): white, amorphous powder, ^{1}H NMR ($CDCl_3$, 400 MHz): δ 1.01 (3H, s, Me-18), 1.03 (3H, s, Me-30), 1.23 (3H, s, Me-28), 1.37 (3H, s, Me-29), 1.39 (9H, s, Me-19, Me-26 and Me-27), 1.43 (3H, s, Me-21), 1.88 (1H, dd, J = 14.1, 8.2 Hz, H-15), 2.03 (1H, m, H-8), 2.54 (1H, d, J = 7.1 Hz, H-17), 2.73 (1H, d, J = 13.7 Hz, H-12a), 3.24 (1H, d, J = 13.7 Hz, H-12b), 3.53 (1H, br s, H-10), 4.40 (1H, m, H-16), 5.77 (1H, m, H-6), 5.96 (1H, br s, H-1), 6.67 (1H, d, J = 15.5 Hz, H-23), 7.11 (1H, d, J = 15.5 Hz, H-24); ESI-MS: 537 $[M + Na]^+$, 559 $[M + HCOO^-]^-$.

25-acetoxy-2α,16α,20(R)-trihydroxy-cucurbita-5,23-dien-3,11,22-trione (**19**): white, amorphous powder, ^{1}H NMR ($CDCl_3$, 400 MHz): δ 0.97 (3H, s, Me-18), 1.19 (3H, s, Me-30), 1.25 (3H, s, Me-28), 1.30 (3H, s, Me-29), 1.33 (3H, s, Me-19), 1.41 (3H, s, Me-21), 1.52 (3H, s, Me-26), 1.55 (3H, s, Me-27), 1.85 (1H, dd, J = 12.6, 8.9 Hz, H-15), 2.01 (3H, s, H-CO$\underline{Me}$), 2.44 (1H, d, J = 7.5 Hz, H-17), 2.64 (1H, d, J = 15.0 Hz, H-12a), 2.86 (1H, br d, J = 10.0 Hz, H-10), 3.16 (1H, d, J = 15.0 Hz, H-12b), 4.05 (1H, dd, J = 12.2, 6.2 Hz, H-2), 4.36 (1H, dd, J = 7.5, 7.0 Hz, H-16), 5.78 (1H, br s, H-6), 6.44 (1H, d, J = 15.7 Hz, H-23), 7.04 (1H, d, J = 15.7 Hz, H-24); ESI-MS: 581 $[M + Na]^+$, 603 $[M + HCOO^-]^-$.

25-acetoxy-3β,16α,20(R)-trihydroxy-cucurbita-5,23-dien-2,11,22-trione (**20**): white, amorphous powder, ^{1}H NMR ($CDCl_3$, 400 MHz): δ 0.85 (3H, s, Me-28), 0.97 (3H, s, Me-18), 1.08 (3H, s, Me-30), 1.32 (3H, s, Me-29), 1.39 (3H, s, Me-19), 1.42 (3H, s, Me-21), 1.54 (3H, s, Me-26), 1.57 (3H, s, Me-27), 1.88 (1H, m, H-15), 2.01 (3H, s, H-CO$\underline{Me}$), 2.48 (1H, d, J = 7.3 Hz, H-17), 2.65 (1H, d, J = 15.3 Hz, H-12a), 2.97 (1H, br d, J = 12.2 Hz, H-10), 3.12 (1H, d, J = 15.3 Hz, H-12b), 3.45 (1H, br s, OH-16), 3.50 (1H, d, J = 2.3 Hz, OH-3), 4.12 (1H, d, J = 2.3 Hz, H-3), 4.27 (1H, s, OH-20), 4.36 (1H, m, H-16), 5.90 (1H, m, H-6), 6.45 (1H, d, J = 15.8 Hz, H-23), 7.05 (1H, d, J = 15.8 Hz, H-24); ^{13}C NMR ($CDCl_3$, 150 MHz): δ 36.4 (C-1), 210.9 (C-2), 79.5 (C-3), 40.9 (C-4), 140.1 (C-5), 122.0 (C-6), 23.8 (C-7), 42.5 (C-8), 48.0 (C-9), 32.4 (C-10), 212.4 (C-11), 48.7 (C-12), 50.5 (C-13), 48.2 (C-14), 45.6 (C-15), 71.4 (C-16), 58.2 (C-17), 19.0 (C-18), 18.5 (C-19), 78.2 (C-20), 24.0 (C-21), 202.4 (C-22), 120.4 (C-23), 151.9 (C-24), 79.3 (C-25), 25.9 (C-26), 26.4 (C-27), 24.4 (C-28), 27.7 (C-29), 19.9 (C-30), 170.2 ($\underline{C}$OMe), 21.9 (CO$\underline{Me}$); ESI-MS: 581 $[M + Na]^+$, 603 $[M + HCOO^-]^-$.

3β,16α,17-trihydroxy-*ent*-kauran-18-yl acetate (**22**): white, amorphous powder; ^{1}H NMR (CD_3OD, 400 MHz): δ 1.02 (3H, s, Me-19), 1.07 (3H, s, Me-20), 2.03 (3H, s, $\underline{CH_3}$CO), 3.59 (1H, d, J = 11.4 Hz, H-17a), 3.61 (1H, dd, J = 3.8, 2.7 Hz, H-3), 3.70 (1H, d, J = 11.4 Hz, H-17b), 3.93 (1H, d, J = 11.3 Hz, H-18a), 4.22 (1H, d, J = 11.3 Hz, H-18b); ESI-MS: 403 $[M + Na]^+$.

16α,17-dihydroxy-*ent*-kauran-3-one (**23**): white, amorphous powder; ^{1}H NMR ($CDCl_3$, 400 MHz): δ 1.02 (3H, s, Me-18), 1.06 (6H, s, Me-19 and Me-20), 3.67 (1H, d, J = 10.1 Hz, H-17a), 3.77 (1H, d, J = 10.1 Hz, H-17b); ESI-MS: 321 $[M + H]^+$, 343 $[M + Na]^+$.

3α,16α,17-trihydroxy-*ent*-kauran (**24**): white, amorphous powder; ^{1}H NMR (CD_3OD, 400 MHz): δ 0.83 (3H, s, Me-18), 0.91 (3H, s, Me-19), 1.07 (3H, s, Me-20), 1.11 (1H, d, J = 5.5 Hz, H-9), 3.32 (1H, dd, overlapped), 3.59 (1H, d, J = 11.3 Hz, H-17a), 3.71 (1H, d, J = 11.3 Hz, H-17b); ^{13}C NMR (CD_3OD, 150 MHz): δ 34.6 (C-1), 26.3 (C-2), 76.8 (C-3), 38.2 (C-4), 50.0 (C-5), 21.1 (C-6), 43.2 (C-7), 45.8 (C-8), 58.0 (C-9), 40.3 (C-10), 19.3 (C-11), 27.3 (C-12), 46.5 (C-13), 38.5 (C-14), 54.1 (C-15), 82.8 (C-16), 66.9 (C-17), 22.6 (C-18), 29.1 (C-19), 18.3 (C-20); ESI-MS: 345 $[M + Na]^+$.

16α,17,18-trihydroxy-*ent*-kaur-3-one (**25**): white, amorphous powder, ^{1}H NMR (CD_3OD, 400 MHz): δ 1.12 (3H, s, Me-19), 1.24 (3H, s, Me-20), 1.95 (1H, br d, J = 11.8 Hz, H-14), 2.06 (1H, br m, H-13), 2.13 (1H, m, H-1), 2.33 (1H, m, H-2b), 2.71 (1H, m, H-2a), 3.50 (1H, d, J = 11.2 Hz, H-17b), 3.59 (1H, d, J = 11.0 Hz, H-18b), 3.70 (1H, d, J = 11.0 Hz, H-18a), 3.98 (1H, d, J = 11.2 Hz, H-17a); ESI-MS: 371 $[M + Cl]^-$, 381 $[M + HCOO^-]^-$.

Figure S1. ^{1}H NMR spectrum of compound **1** in CD_3OD (600 MHz)

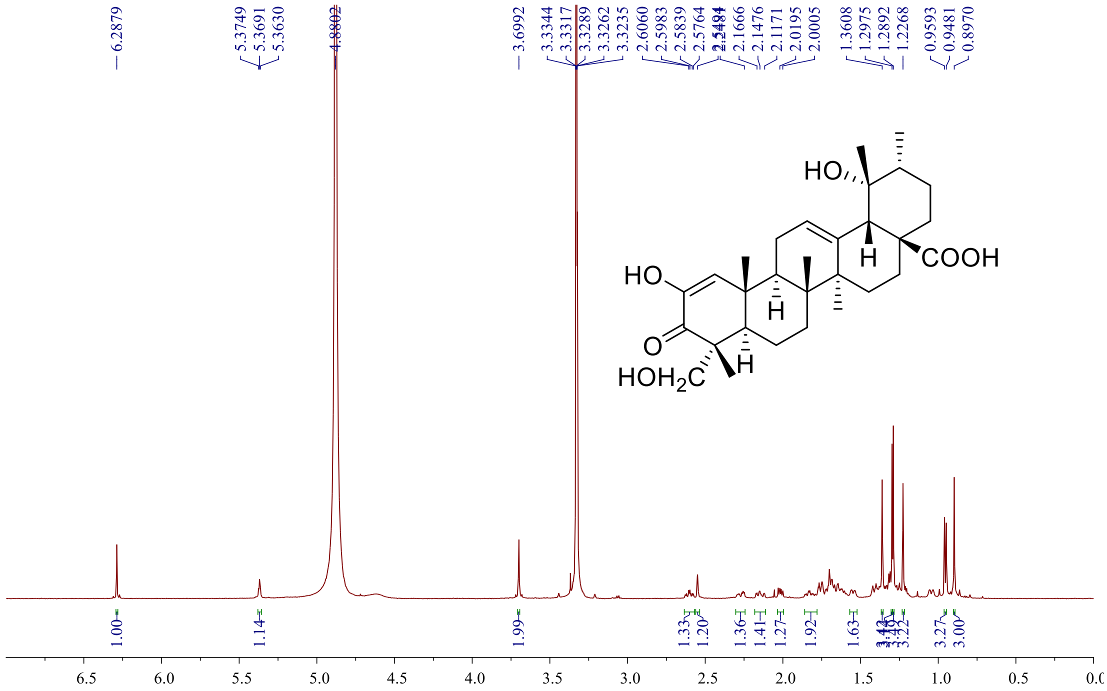

Figure S2. ^{13}C NMR spectrum of compound **1** in CD_3OD (150 MHz)

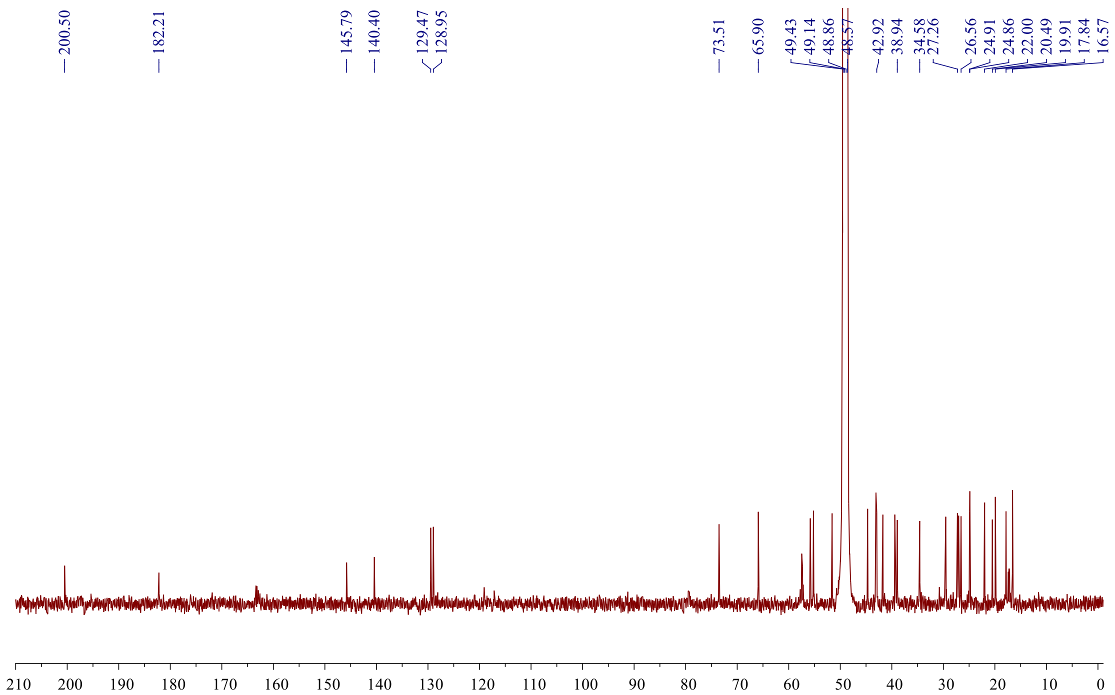

Figure S3. HMBC spectrum of compound **1** in CD_3OD (600 MHz)

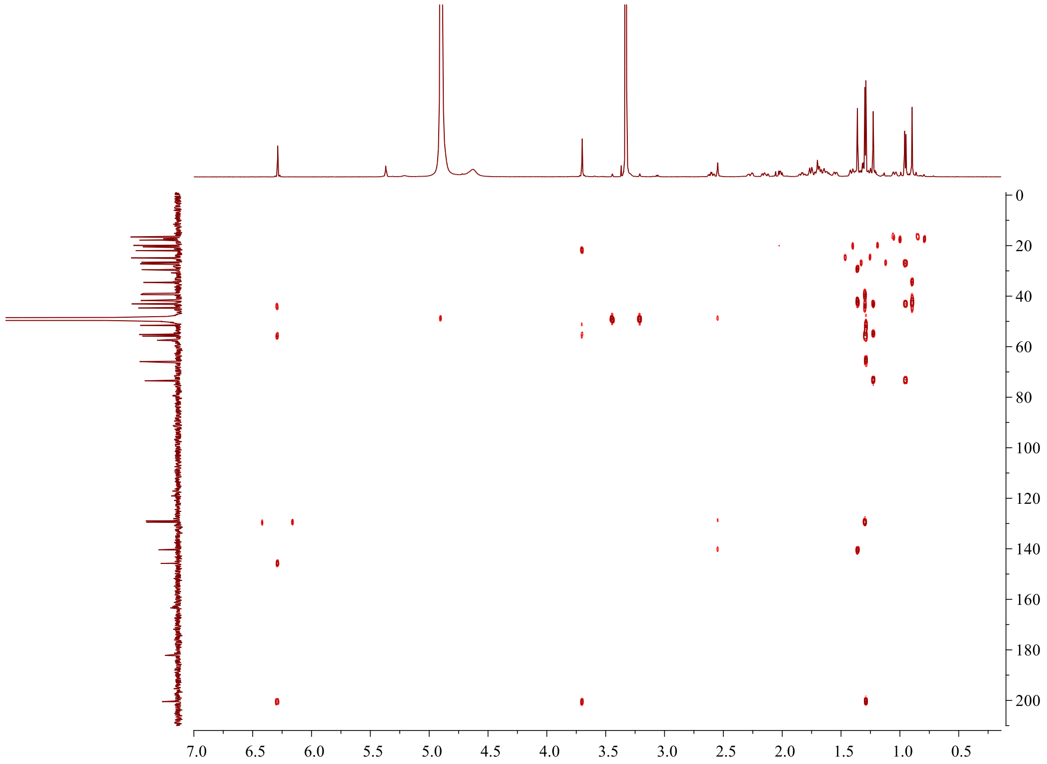

Figure S4. ROESY spectrum of compound **1** in CD_3OD (600 MHz)

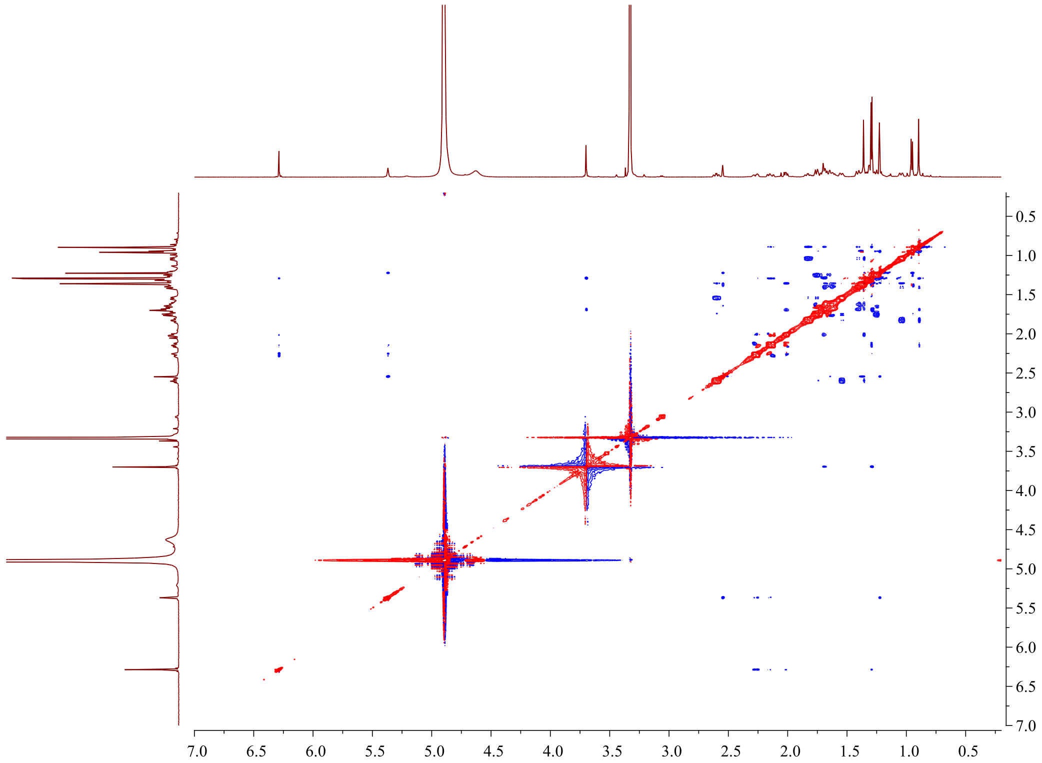

Figure S5. HRESIMS report of compound **1**.

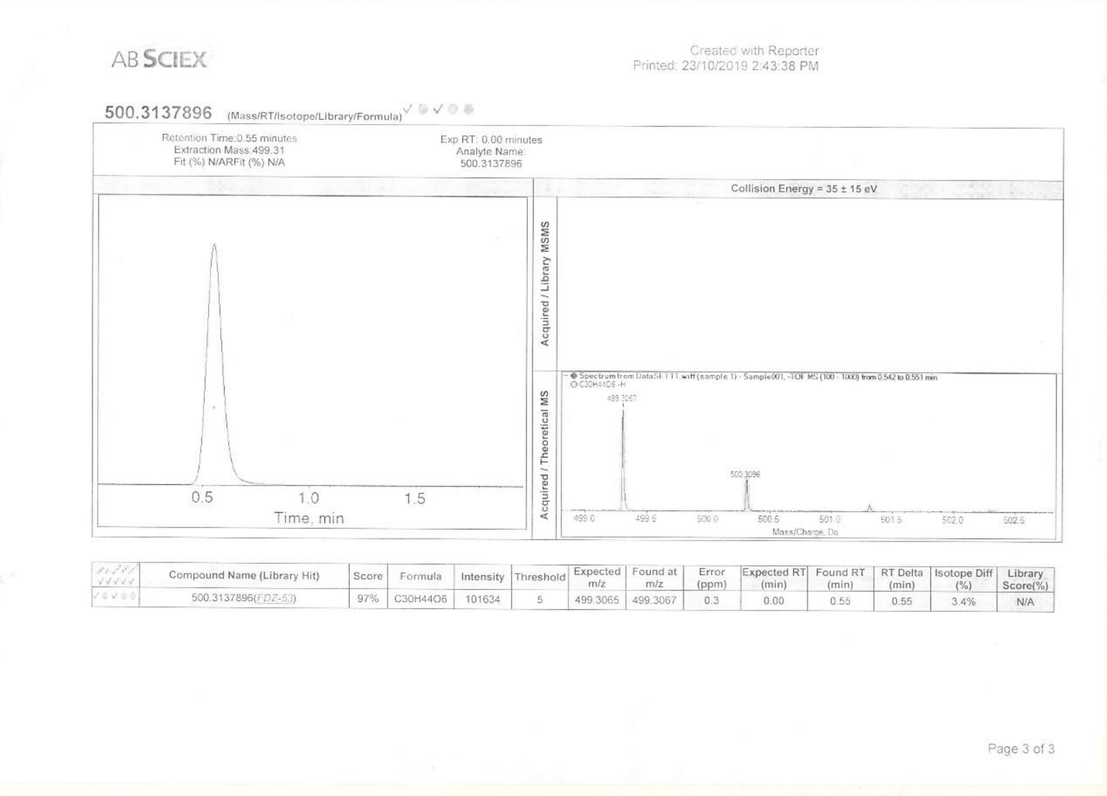

AB SCIEX

Created with Reporter
Printed: 23/10/2019 2:43:38 PM

500.3137896 (Mass/RT/Isotope/Library/Formula)

Retention Time:0.55 minutes
Extraction Mass:499.31
Fit (%) N/ARFit (%) N/A

Exp RT: 0.00 minutes
Analyte Name:
500.3137896

Collision Energy = 35 ± 15 eV

	Compound Name (Library Hit)	Score	Formula	Intensity	Threshold	Expected m/z	Found at m/z	Error (ppm)	Expected RT (min)	Found RT (min)	RT Delta (min)	Isotope Diff (%)	Library Score(%)
	500.3137896(FDZ-53)	97%	C30H44O6	101634	5	499.3065	499.3067	0.3	0.00	0.55	0.55	3.4%	N/A

Page 3 of 3

Figure S6. Experimental CD spectrum of compound **1** in MeOH.

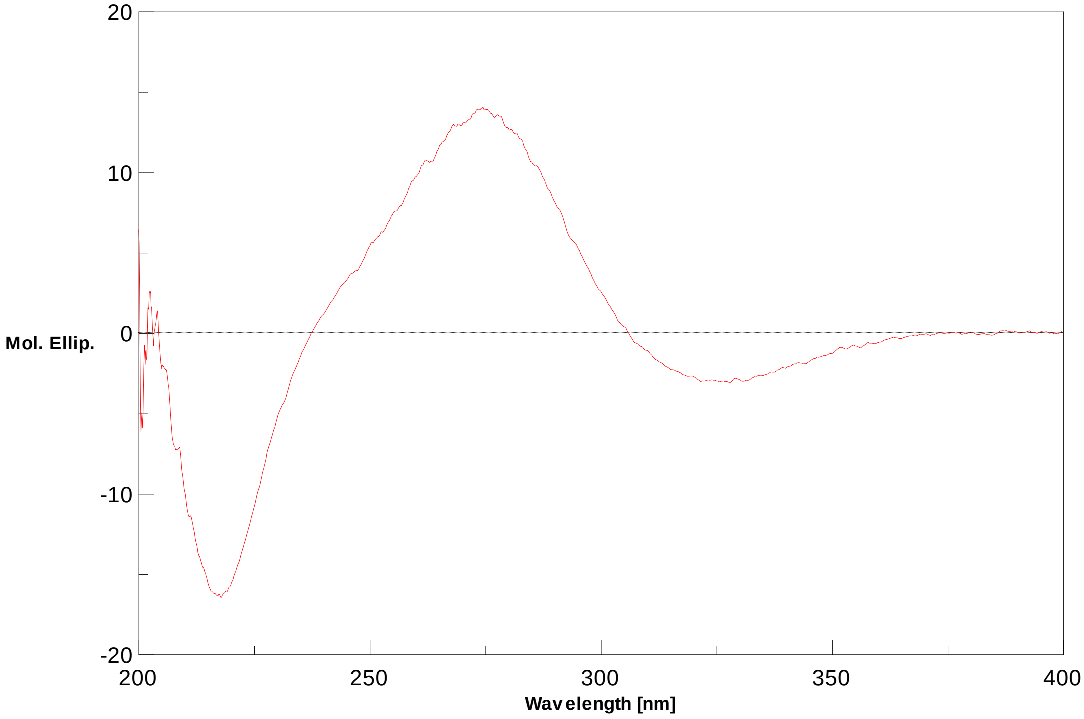

Figure S7. ^{1}H NMR spectrum of compound **2** in CD_3OD (600 MHz)

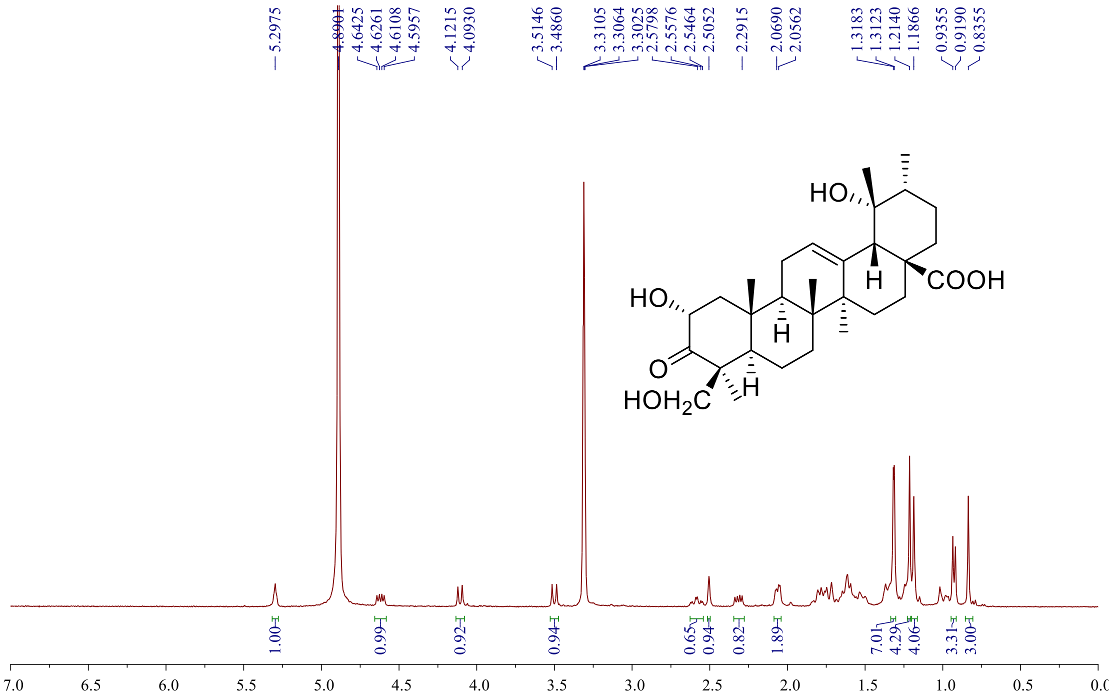

Figure S8. ^{13}C NMR spectrum of compound **2** in CD_3OD (150 MHz)

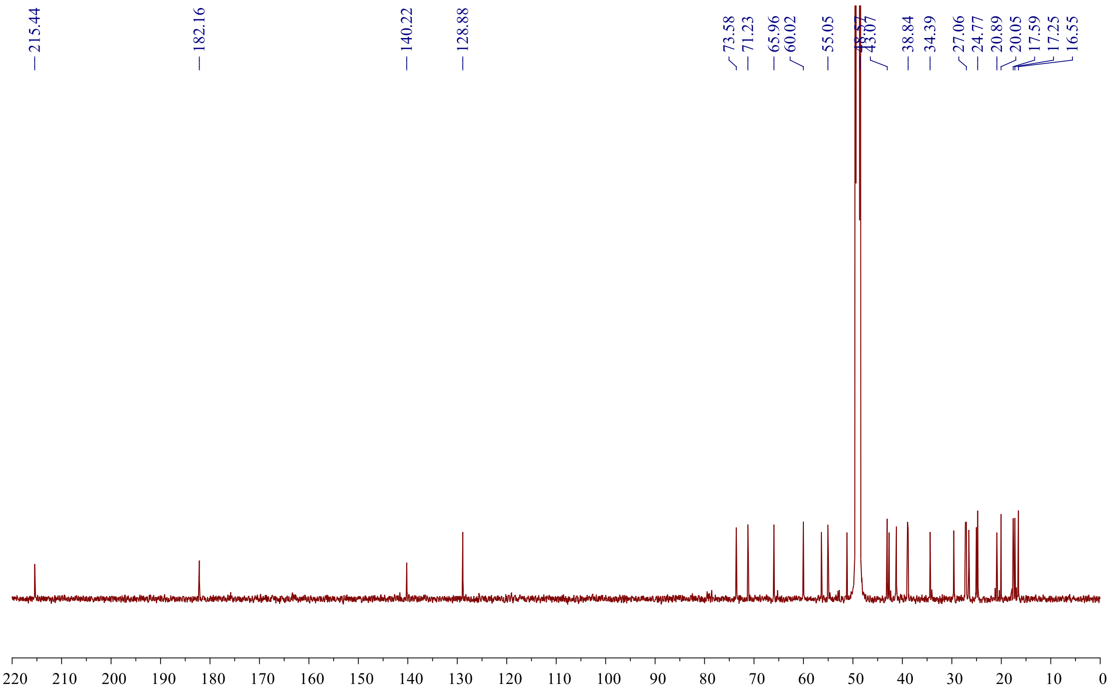

Figure S9. HMBC spectrum of compound **2** in CD3OD (600 MHz)

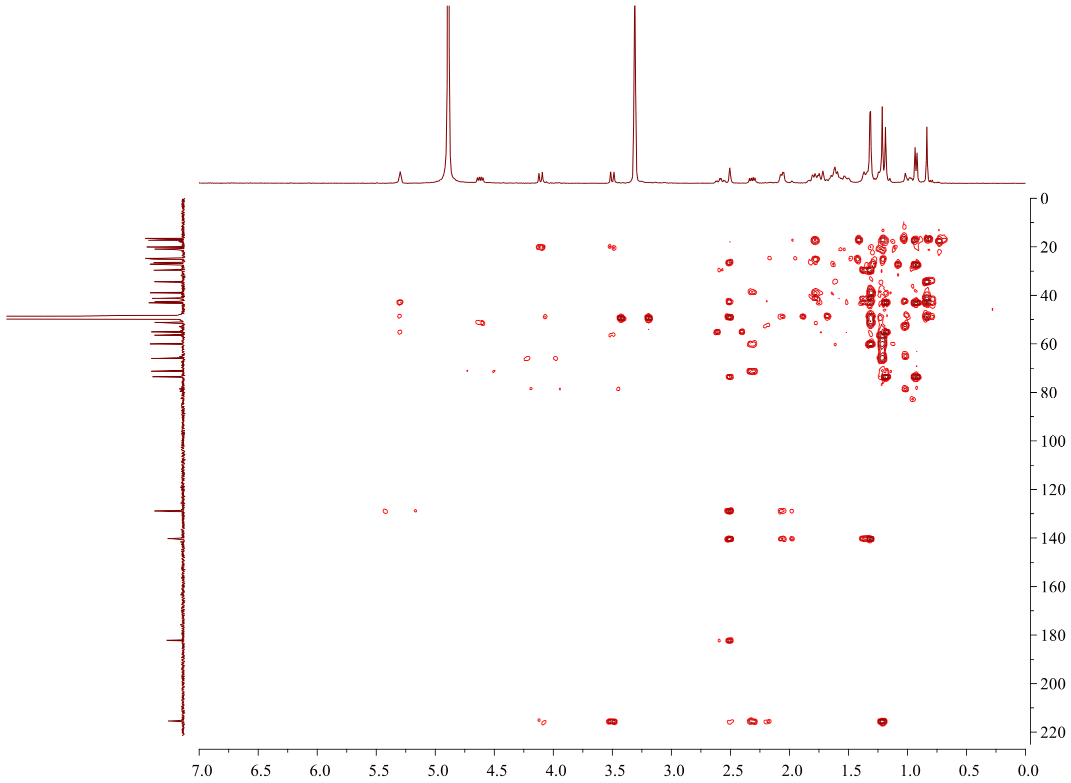

Figure S10. ROESY spectrum of compound **2** in CD3OD (600 MHz)

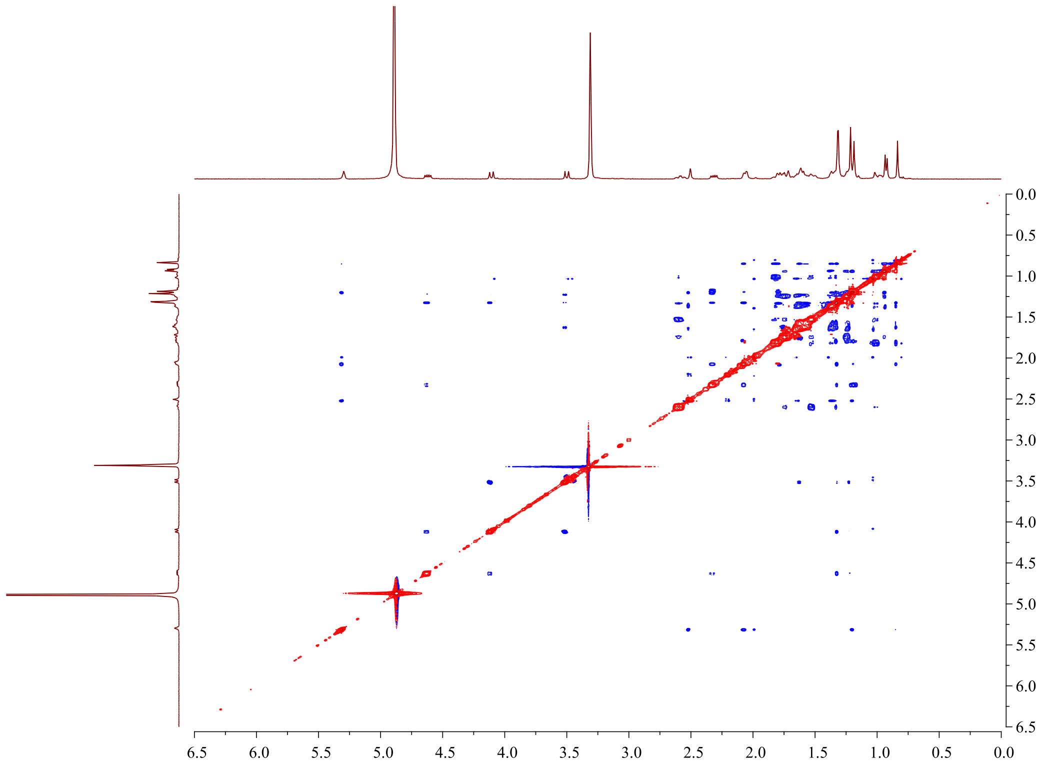

Figure S11. HRESIMS report of compound **2**.

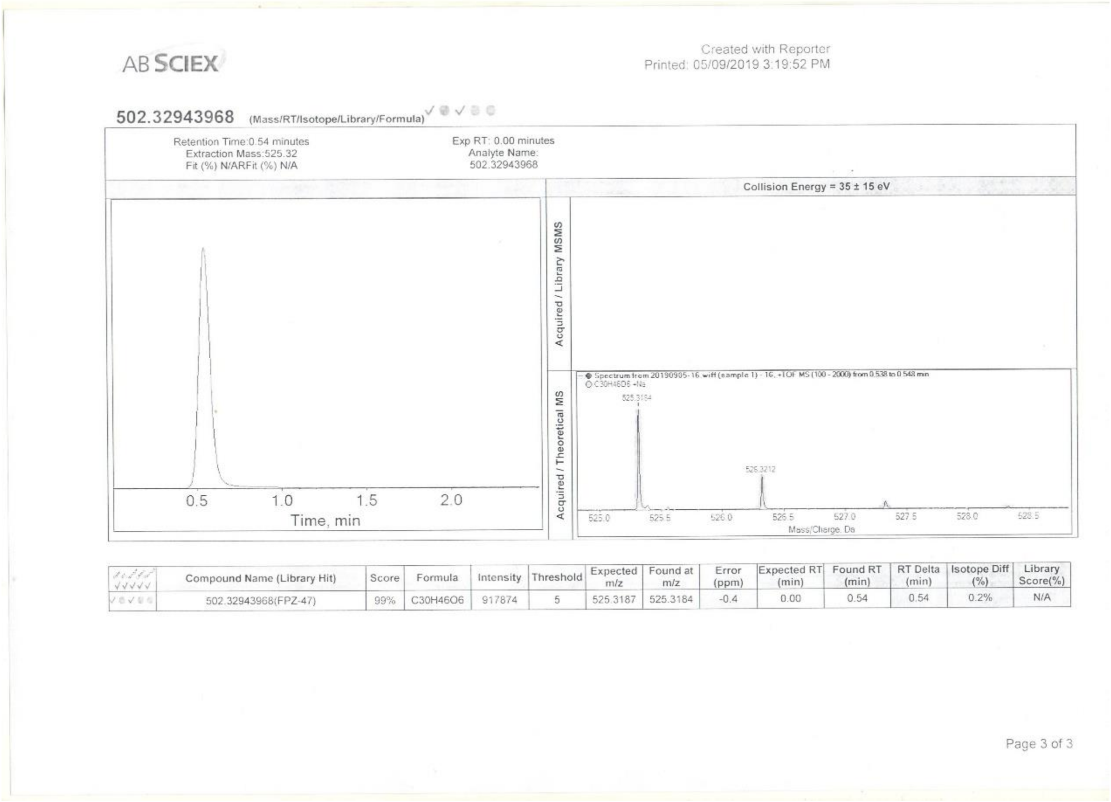

	Compound Name (Library Hit)	Score	Formula	Intensity	Threshold	Expected m/z	Found at m/z	Error (ppm)	Expected RT (min)	Found RT (min)	RT Delta (min)	Isotope Diff (%)	Library Score(%)
	502.32943968(FPZ-47)	99%	C30H46O6	917874	5	525.3187	525.3184	-0.4	0.00	0.54	0.54	0.2%	N/A

Figure S12. ^1H NMR spectrum of compound **3** in CD_3OD (600 MHz)

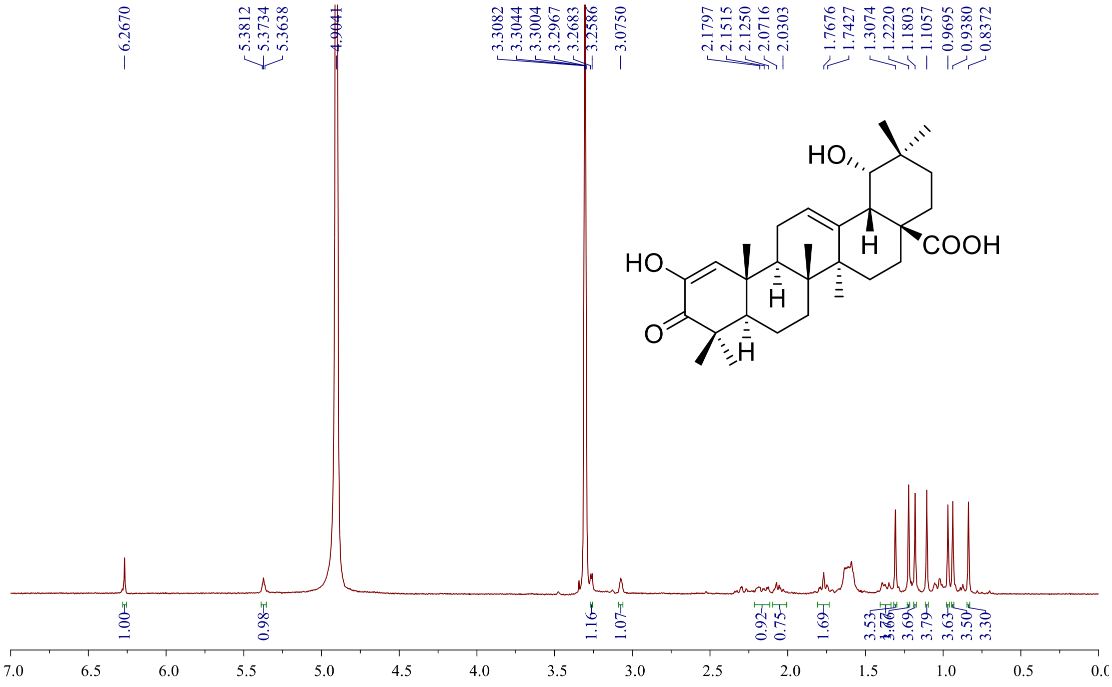

Figure S13. ^{13}C NMR spectrum of compound **3** in CD3OD (150 MHz)

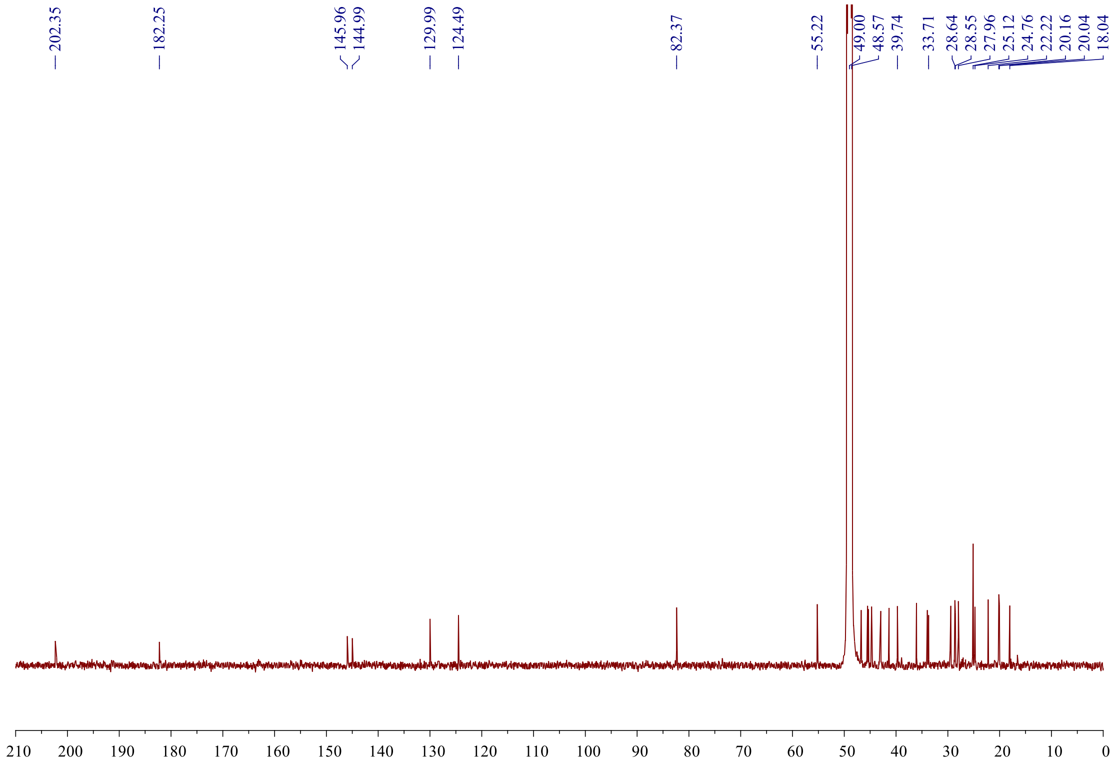

Figure S14. ^{1}H-^{1}H COSY spectrum of compound **3** in CD3OD (600 MHz)

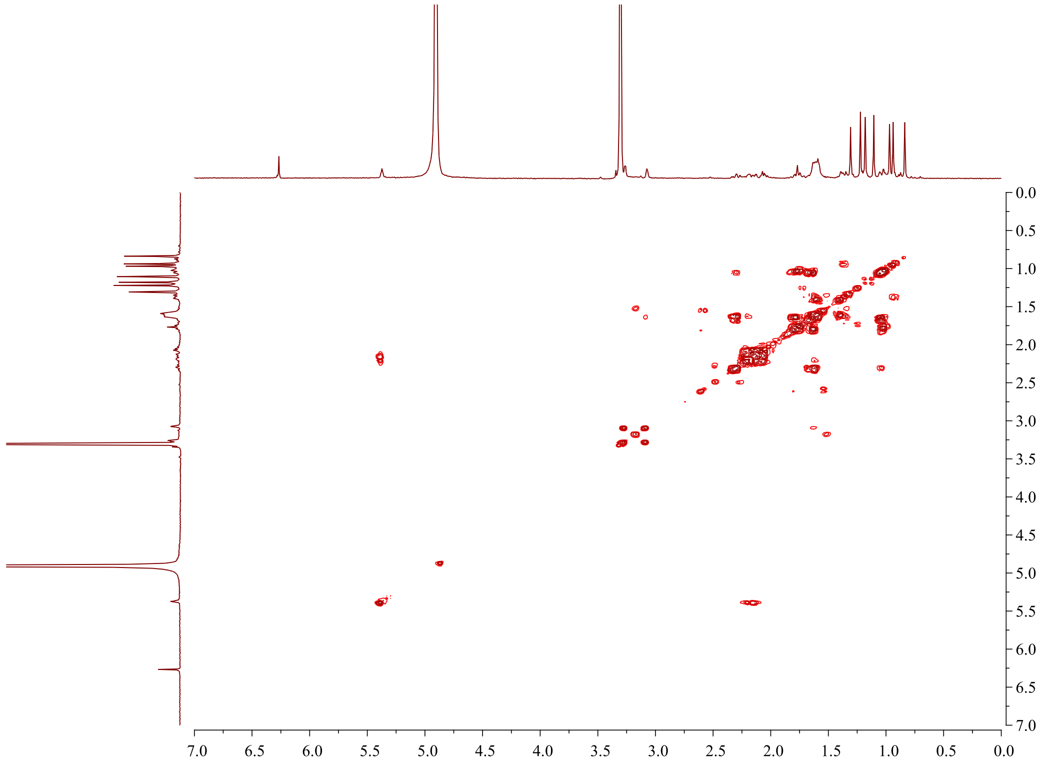

Figure S15. HMBC spectrum of compound **3** in CD_3OD (600 MHz)

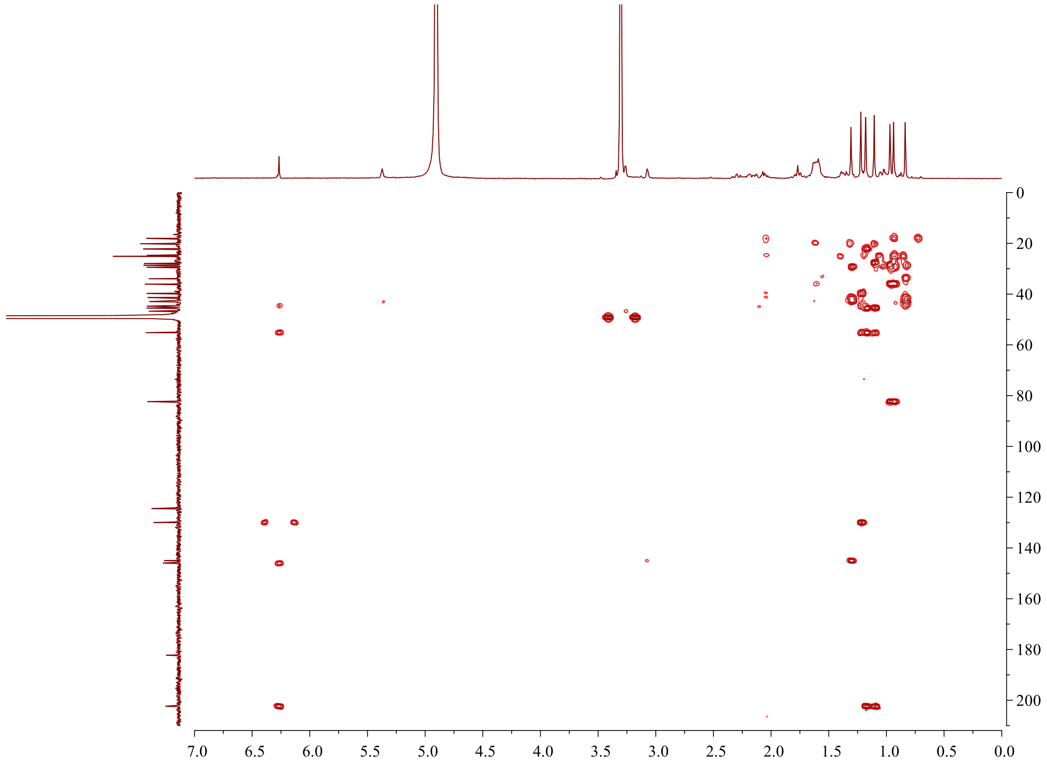

Figure S16. ROESY spectrum of compound **3** in CD_3OD (600 MHz)

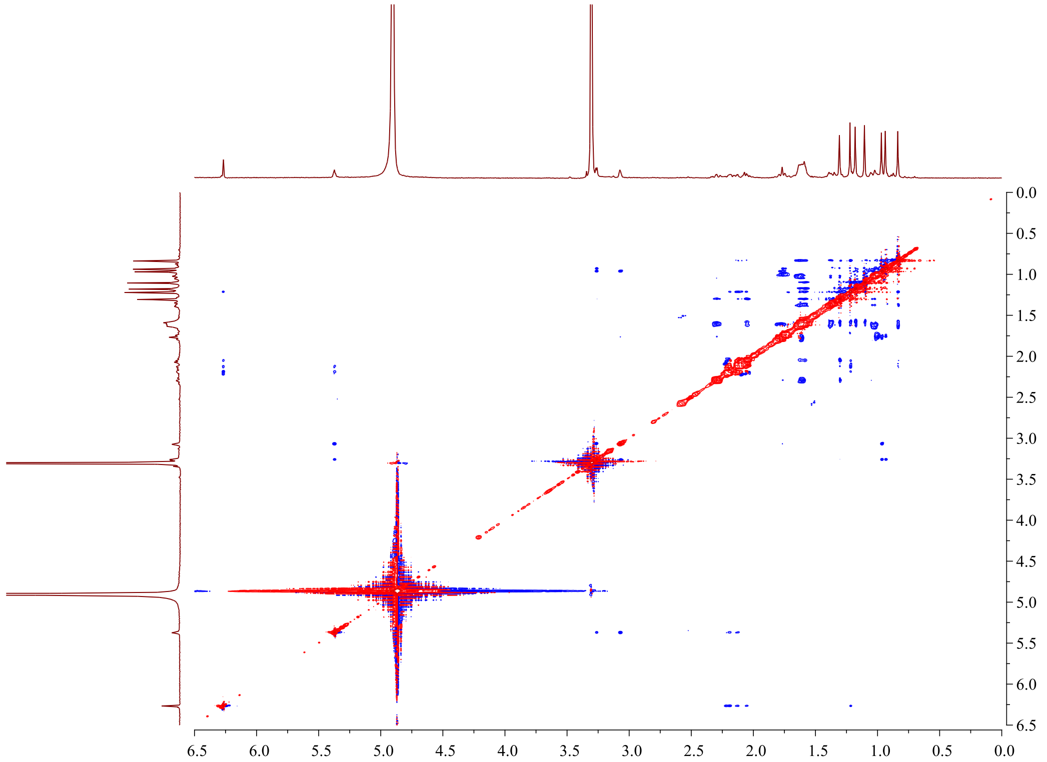

Figure S17. HRESIMS report of compound **3**.

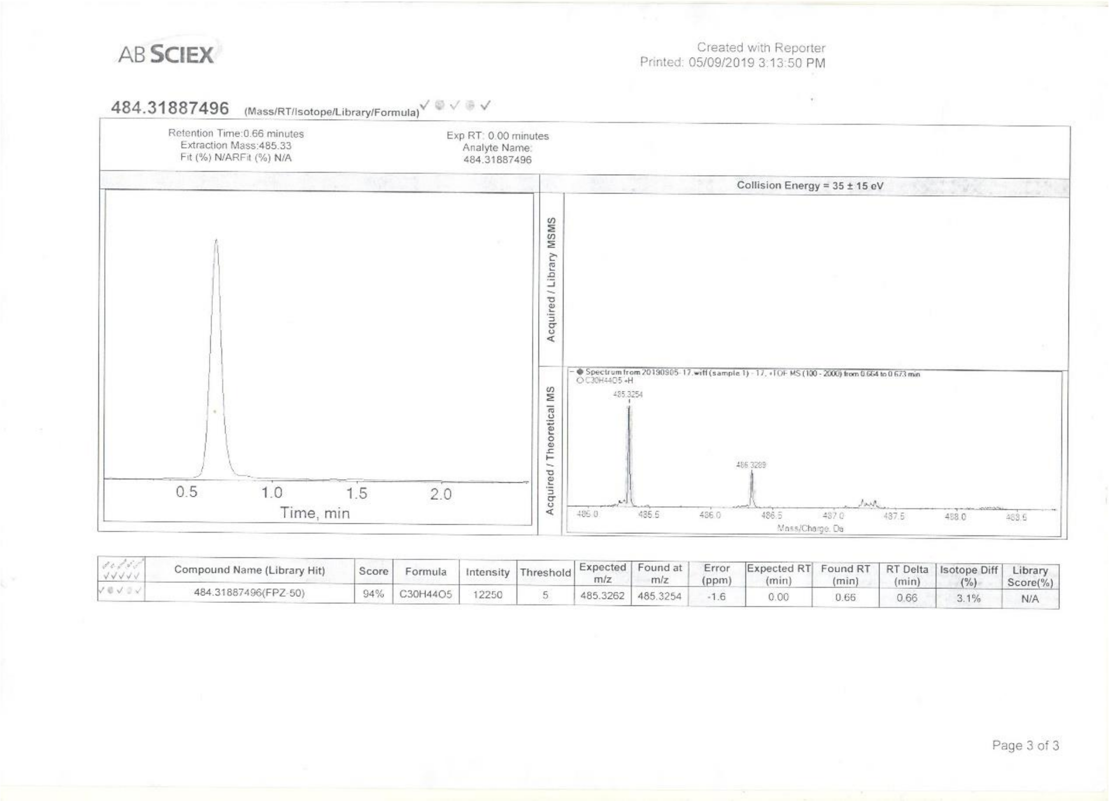

AB SCIEX

Created with Reporter
Printed: 05/09/2019 3:13:50 PM

484.31887496 (Mass/RT/Isotope/Library/Formula)

Retention Time:0.66 minutes
Extraction Mass:485.33
Fit (%) N/ARFit (%) N/A

Exp RT: 0.00 minutes
Analyte Name:
484.31887496

Collision Energy = 35 ± 15 eV

	Compound Name (Library Hit)	Score	Formula	Intensity	Threshold	Expected m/z	Found at m/z	Error (ppm)	Expected RT (min)	Found RT (min)	RT Delta (min)	Isotope Diff (%)	Library Score(%)
	484.31887496(FPZ-50)	94%	C30H44O5	12250	5	485.3262	485.3254	-1.6	0.00	0.66	0.66	3.1%	N/A

Page 3 of 3

Figure S18. Experimental CD spectrum of compound **3** in MeOH.

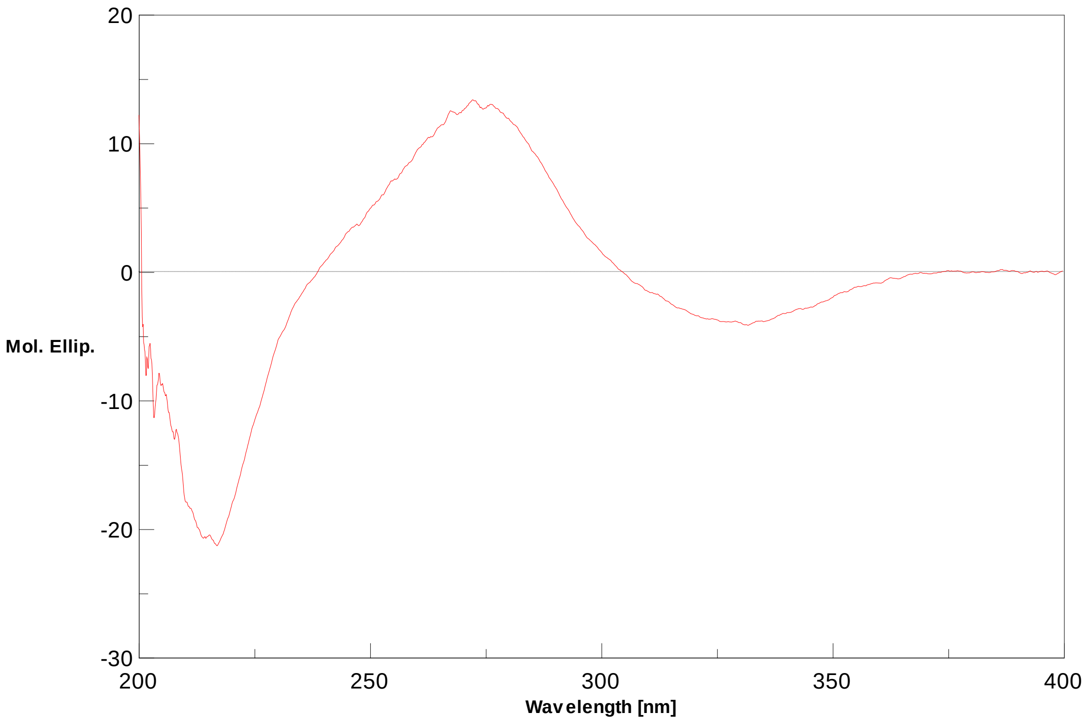

Figure S19. ^{1}H NMR spectrum of compound **21** in C_6D_6 (600 MHz)

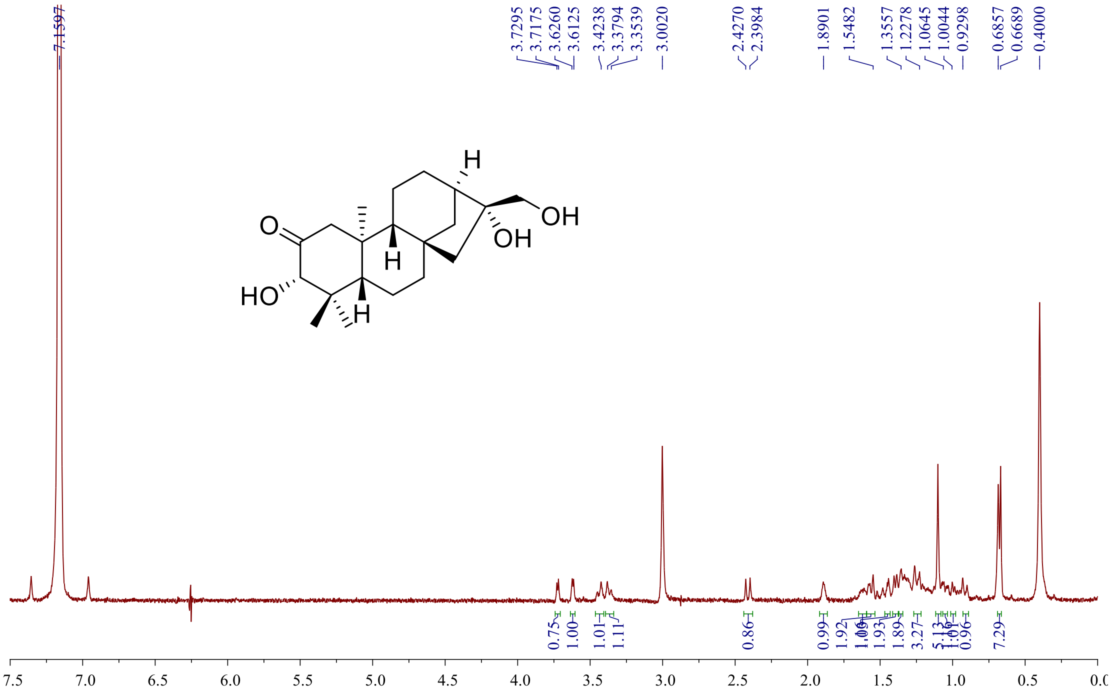

Figure S20. ^{13}C NMR spectrum of compound **21** in C_6D_6 (150 MHz)

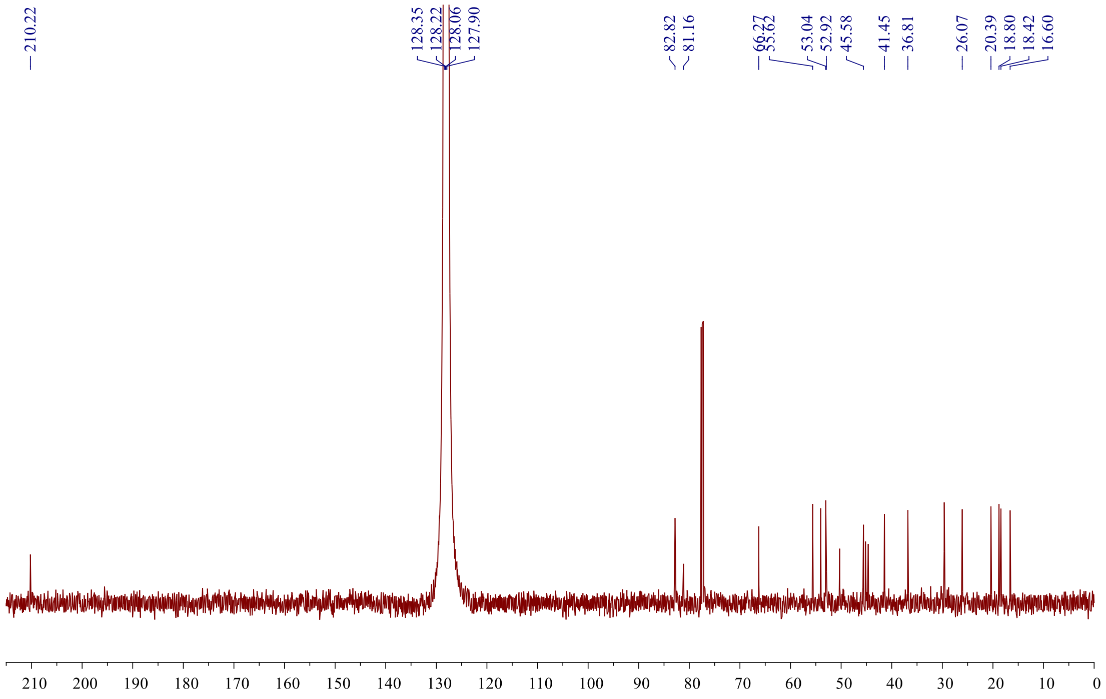

Figure S21. ^{1}H NMR spectrum of compound **21** in C_5D_5N (600 MHz)

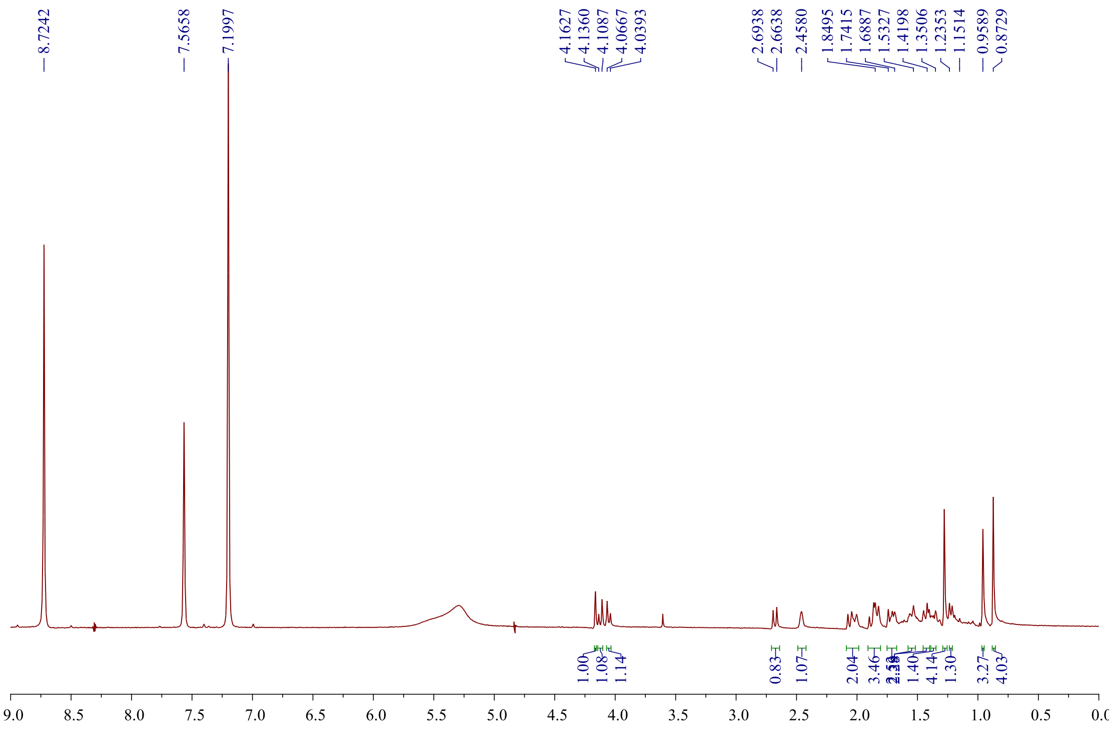

Figure S22. NOESY spectrum of compound **21** in C_5D_5N (600 MHz)

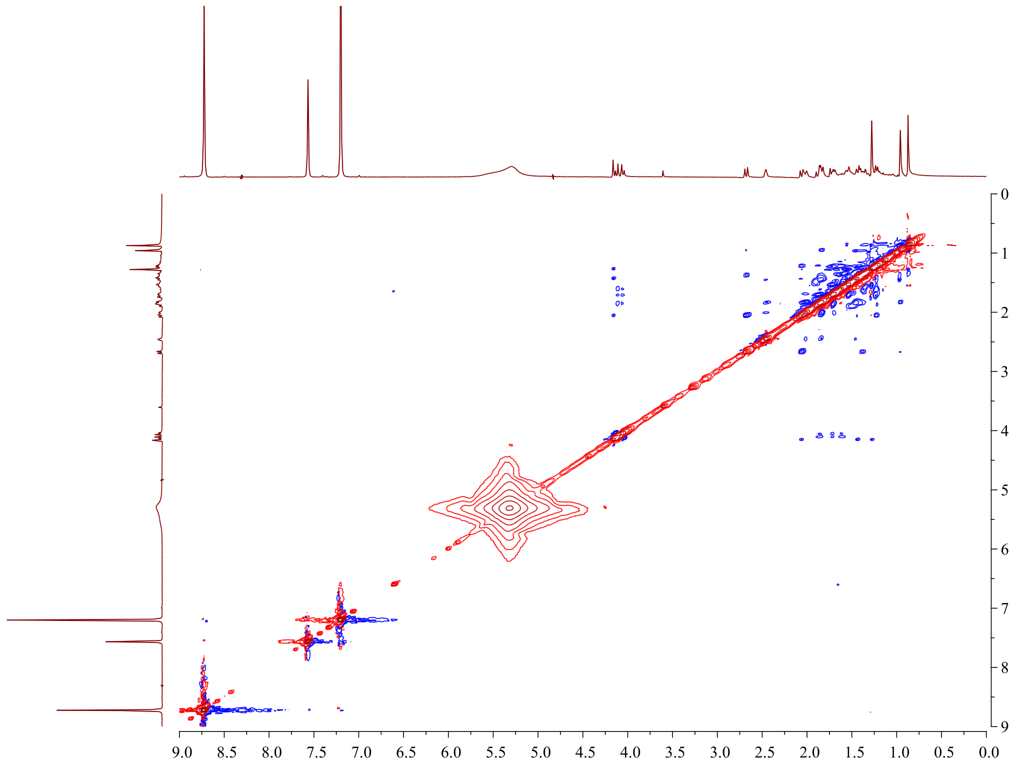

Figure S23. HRESIMS report of compound **21**.

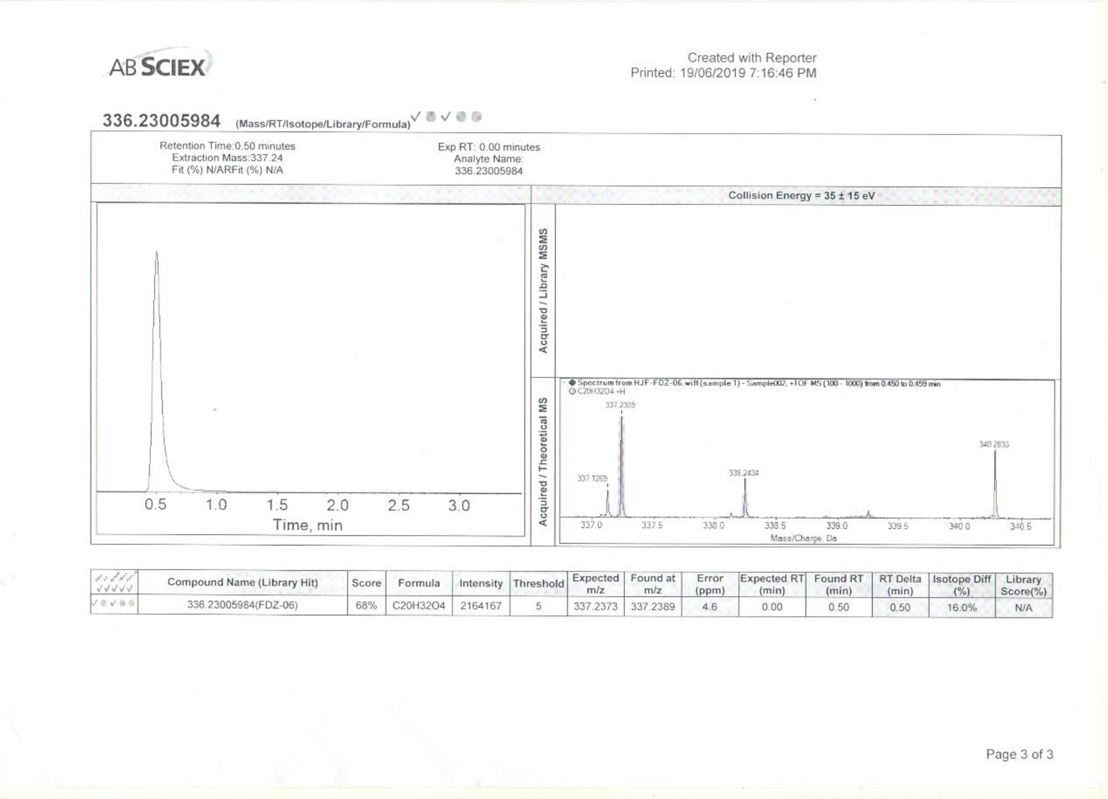
AB SCIEX

Created with Reporter
Printed: 19/06/2019 7:16:46 PM

336.23005984 (Mass/RT/Isotope/Library/Formula)

Retention Time:0.50 minutes
Extraction Mass:337.24
Fit (%) N/ARFit (%) N/A

Exp RT: 0.00 minutes
Analyte Name:
336.23005984

Collision Energy = 35 ± 15 eV

	Compound Name (Library Hit)	Score	Formula	Intensity	Threshold	Expected m/z	Found at m/z	Error (ppm)	Expected RT (min)	Found RT (min)	RT Delta (min)	Isotope Diff (%)	Library Score(%)
	336.23005984(FDZ-06)	68%	C20H32O4	2164167	5	337.2373	337.2389	4.6	0.00	0.50	0.50	16.0%	N/A

Page 3 of 3

Figure S24. Experimental CD spectrum of compound **21** in MeOH.

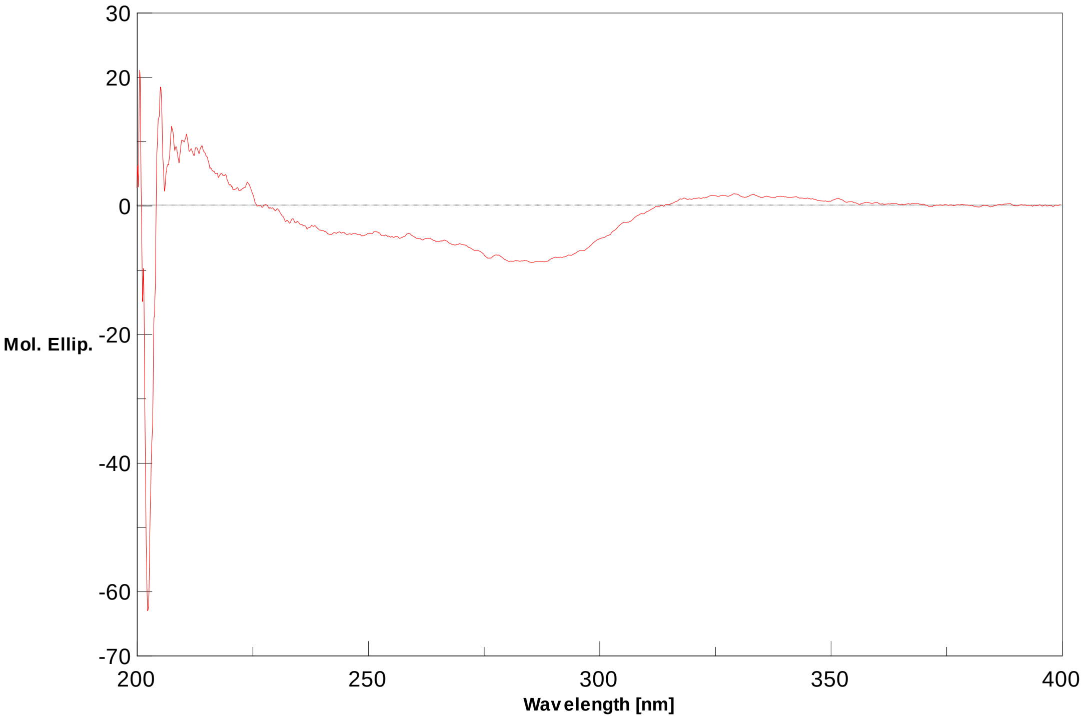

Supplement: Supplementary file 1 [file molecules-26-01911-s001.pdf]
